# Debonding characteristics of innovative 3D-printed lingual retainers following artificial aging and acid challenge

**DOI:** 10.1590/2177-6709.30.6.e2425264.oar

**Published:** 2026-01-23

**Authors:** Noor Salam ALNUAIMY, Akram Faisal ALHUWAIZI, Akram Mohammed ALTAMIMI

**Affiliations:** 1 Al-Kunooze University, College of Dentistry, Department of Orthodontics (Albasrah, Iraq). Al-Kunooze University College of Dentistry Department of Orthodontics Albasrah Iraq; 2 University of Baghdad, College of Dentistry, Department of Orthodontics (Baghdad, Iraq). University of Baghdad College of Dentistry Department of Orthodontics Baghdad Iraq; 3 Al-Kunooze University, College of Dentistry, Department of Restorative Dentistry (Albasrah, Iraq). Al-Kunooze University College of Dentistry Department of Restorative Dentistry Albasrah Iraq

**Keywords:** Artificial aging, Cyclic loading, Lingual retainer, Nanofiller, 3D-printed retainer, Envelhecimento artificial, Carga cíclica, Aparelho de contenção lingual, Nanopartículas de preenchimento, Aparelho de contenção impresso em 3D

## Abstract

**Introduction:**

Traditional fixed retainers used after orthodontic therapy have many downsides.

**Objective:**

Innovative 3D-printed fixed retainers have been manufactured using nanoceramic hybrid resin-based material (SprintRay OnX^®^), investigating their durability to support long-term oral functions.

**Methods:**

One hundred acrylic blocks with mounted premolar pairs were allocated into five groups, including three groups of 3D-printed retainers with 1-mm round cross-section, one group with 1.5-mm oval cross-section, and one group with 1.5-mm semi-elliptical cross-section, and conventional multistranded stainless steel G&H^®^ retainers and Respond^®^ retainers. The models with bonded retainers were exposed to artificial aging or acid challenge. The debonding forces and the subsequent failure patterns were investigated.

**Results:**

The 3D-printed semi-elliptical retainers exhibited the greatest debonding forces, while the 3D-printed oval, G&H®, 3D-printed round, and Respond^®^ retainers decreased in descending order. After the acid challenge, no statistically significant differences were observed between the 3D-printed semi-elliptical and oval retainers. The 3D-printed retainers showed a prevailing cohesive fracture.

**Conclusion:**

The debonding forces of 3D-printed semi-elliptical and oval retainers following simulated aging and acid exposure were encouraging. They might be a good candidate as lingual retainers.

## RESUMO

**Introdução:**

Os aparelhos de contenção fixos tradicionais usados após o tratamento ortodôntico apresentam muitas desvantagens.

**Objetivo:**

Aparelhos de contenção fixos inovadores, impressos em 3D, foram fabricados utilizando um material híbrido à base de resina nanocêramica (SprintRay OnX^®^), para investigar sua durabilidade para suportar funções orais no longo prazo.

**Métodos:**

Cem blocos de acrílico com pares de pré-molares montados foram alocados em cinco grupos, incluindo três grupos de contenções impressas em 3D com secção transversal circular de 1 mm, um grupo com secção transversal oval de 1,5 mm e um grupo com secção transversal semielíptica de 1,5 mm, além de contenções convencionais G&H^®^ e Respond^®^ com fio de aço inoxidável multifilamentado. Os modelos com contenções coladas foram submetidos a envelhecimento artificial ou ataque ácido. As forças de descolagem e os padrões de falha subsequentes foram investigados.

**Resultados:**

As contenções semielípticas impressas em 3D apresentaram as maiores forças de descolagem, enquanto as contenções ovais impressas em 3D, G&H®, circular impressa em 3D e Respond® apresentaram resistência decrescente, nessa ordem. Após o ataque ácido, não foram observadas diferenças estatisticamente significativas entre as contenções semielípticas e ovais impressas em 3D. As contenções impressas em 3D apresentaram predominantemente fratura coesiva.

**Conclusão:**

As forças de descolagem das contenções semielípticas e ovais impressas em 3D após envelhecimento simulado e ataque ácido foram promissoras. Elas podem ser boas candidatas para uso como contenções linguais.

## INTRODUCTION

The traditional stainless steel multistranded wire (MSW) retainer is still largely considered the gold standard retainer.^1^^,^^2^ However, this retainer faces many challenges, such as a high rate of failure, negative impact on the maintenance of periodontal health, undesirable dental movements, and esthetic issues.^3^ This retainer is also not recommended for individuals with metal hypersensitivity and must be removed before a magnetic resonance imaging (MRI) scan.^4^ CAD/CAM customized fixed retainers using milling techniques have been introduced recently, promising greater success.^2^ However, these retainers do not appear to be clinically more effective than MSWs.^5^ They continue to have limitations, such as bonding difficulties, increased rate of failure, dependence on an external facility, and relatively high costs.^2^^,^^6^^-^^8^

The 3D printing is a cutting-edge technology in the manufacturing industry.^9^ The clinical orthodontic discipline has become more efficient due to the recent rapid advances in digital and 3D printing technologies offering cost-efficiency, lesser wasting materials, fabrication speed, superior quality and accuracy, and intricate object production.^10^^,^^11^ However, ordinary resin 3D-printed products still have some drawbacks related to their physical and mechanical properties. Incorporating nanoparticles or nanofillers into the raw material, utilizing their unique properties, including reactivity and high surface area, may push the boundaries of 3D printing technologies, facilitating the development of multifunctional materials with customized qualities.^12^^,^^13^

The present study sought to create innovative, 3D-printed personalized fixed retainers from nanoceramic hybrid resin-based material. This nanomaterial was used to fabricate prosthetic intraoral devices. It is a resin packed in nanoceramics to give it phenomenal strength and esthetic, a critical consideration when fabricating lingual retainer. The proposed benefits may include improved fitting accuracy, position, and interproximal adaptation, which may prevent direct occlusal contact with lower teeth, thereby reducing the chance of failure of the maxillary retainer. Passive fit is also essential, as the tension of the wire during bonding may result in unanticipated teeth movements. It is suitable for individuals with metal hypersensitivity or for MRI scans, due to its complete absence of metals. The objectives of this study were to measure the debonding forces and failure patterns after artificial aging and acid challenge protocols used to mimic the exposure of bonded retainers to intraoral challenges.

## MATERIAL AND METHODS

### PREPARATION OF SAMPLES

Ethical approval (Ref. number: 619) was obtained from the local Research Ethical Committee. Maxillary first premolars (245 teeth) were gathered from patients aged 15 to 30 years, which were extracted for orthodontic needs. The stereomicroscope with a magnification of 10x was used to inspect the teeth. The criteria for selecting the teeth included: intact lingual surfaces with no cracks, restorations or caries, and no previous bleaching or orthodontic treatments. Teeth of average size were selected, with a mesiodistal width of 7 to 7.5 mm, and a length of 22 to 23 mm. The chosen teeth were stored at ambient temperature in a weekly-changed 0.1% thymol solution. Each tooth crown was cut from a single proximal aspect by less than 1 mm, to imitate the close contact between the front teeth.

The analyzing rod of the dental surveyor was used to vertically mount each tooth pair in the acrylic into a cylindrical-shaped plastic mold. The lingual surfaces were aligned at the same level using a specially-made T-shaped instrument affixed to the surveyor. The mixed self-cure acrylic resin was poured 2 mm apically to the levels of cementoenamel junctions. These blocks remained at room temperature in distilled water until they were bonded.

**Figure 1:**
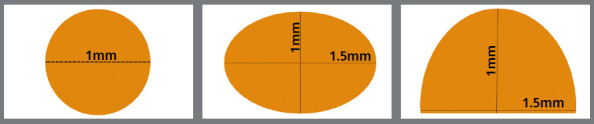
Cross-sections of the 3D-printed customized nanoceramic hybrid lingual retainers.

The sample blocks were grouped into five sets of 20, in the following order (Fig. 1):

» Group I (3D-printed round): 3D-printed customized round fixed retainers with a diameter of 1 mm.
» Group II (3D-printed oval): 3D-printed customized oval fixed retainers with a thickness of 1 mm and a width of 1.5 mm
» Group III (3D-printed semi-elliptical): 3D-printed customized semi-elliptical fixed retainers with a thickness of 1 mm and a width of 1.5 mm.
» Group IV (G&H^®^ MSWs): G&H^®^ multistrand retainer wire; 0.0195-in 7-stranded stainless steel twisted retainers (G&H^®^ Orthodontics, USA).
» Group V (Respond^®^ MSWs): Respond^®^ archwire; 0.0195-in 6-stranded stainless steel dead-soft coaxial retainers (Ormco Corp., USA).

Each group was further divided into 10 specimens subjected to debonding after either artificial aging or acid attack exposure.

**Figure 2:**
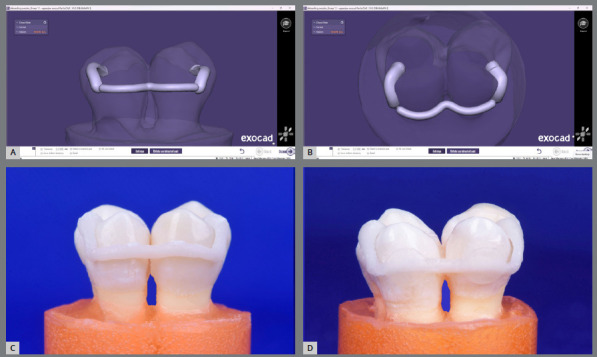
The 3D-printed customized lingual retainer: **A**, **B)** designing; **C)** before bonding; **D)** after bonding.

The intraoral scanner (3Shape TRIOS 3; 3Shape, Denmark) was used to scan the affixed teeth of the 3D-printed groups, which produced STL files. The scanner had been calibrated earlier according to the manufacturer’s specifications. The retainers were designed using Exocad Dental CAD software. The retainer was conformed touching the middle third of the lingual surfaces of teeth, and adaptable to the interdental region. Two attached jigs, adapted to the occlusal and lingual surfaces, were designed distally to both bonding sites to facilitate accurate placement and handling (Fig. 2A, 2B). The Autodesk Meshmixer software was used to re-mesh the design’s STL format, enhancing the printing quality.

The retainers were printed following the manufacturer’s guidelines using the Pro 95 Printer (SprintRay, California, USA) as well as RayWare software (version 2.9.1). The SprintRay OnX nanoceramic hybrid resin-based material container (SprintRay, California, USA) was mixed well with an LC-3D Mixer before each printing process. The retainers were produced horizontally to the building base, to prevent failure due to the irregular fine wire structure, which prohibits vertical print. They were built with a 50-µm layer thickness and remained on the build base for 5-10 minutes. Next, they were cleansed by 91% isopropyl alcohol spraying, and dried using compressed air, to eliminate all residual uncured resin. Seven cleansing cycles were implemented. The retainers were then post-processed using the ProCure Post-Curing System (SprintRay, California, USA), with a 90-watt LED, 28.8 mW/cm^2^ light intensity and 365-405 nm wavelength, for 60 minutes at 60°C, to enhance strength, precision, and polymerization. The supports were cut using a diamond disc as close to the retainer as possible (Fig. 2C). To ensure sufficient print accuracy, the retainers were examined using an 10x magnification stereomicroscope and a digital caliper. Samples with flaws were discarded.

MSWs retainer of 15 mm length was manually gently curved, fitting passively over the middle thirds of the lingual teeth surfaces, close to the interdental region and parallel to the block bases. 

## BONDING METHOD

The lingual teeth surfaces were polished for 10 seconds with pumice free of fluoride, using rubber cups, rinsed with water spray for 10 seconds, and dried with compressed air for 10 seconds. Next, they were etched using Super-Etch etch gel with 37% phosphoric acid (SDI, USA) for 30 seconds, completely rinsed with water for 20 seconds, and dried out using compressed air for 20 seconds, until the area being etched turned chalky white. An even thin primer coating (Transbond^™^ XT; 3M Unitek, California, USA) was put on the lingual surfaces of teeth, and then delicately dispersed with a compressed air jet for 3 seconds. 

A mini-mold wire bonder (4-mm diameter and 1.5-mm depth) was employed to standardize the bond pad’s form and dimensions, and confirm that the wire was accurately centered inside the adhesive bond. In line with the manufacturer’s directions, the adhesive (Transbond^™^ LR; 3M Unitek, California, USA) underwent light curing for 10 seconds using 3M^™^ Elipar^™^ DeepCure-L (3M ESPE, Germany), with a light intensity of 1470 mW/cm^2^ and wavelength of 430-480 nm (Fig. 2D). Next, the retainer jigs were meticulously eliminated with a diamond fine fissure bur.

## DEBONDING FORCES

### A. ARTIFICIAL AGING

Fifty bonded specimens were subjected to a two-year aging protocol, during which retainer failures were reported in the clinical studies.^14^ The thermocycling procedure was applied following ISO/TS.11405^15^ recommendation, to imitate the intraoral thermal fluctuation. Samples were placed inside a perforated stainless steel container of a custom-made thermocycler, and subjected to 20,000 cycles between 55±0.5°C and 5±0.5°C water baths, with 30 seconds dwelling time and 5 seconds transfer time each way.

The thermocycling was followed by a cyclic loading procedure using a custom-made cyclic loading machine, with an automatic cut-off calculator, when the specimen failed. A stainless steel chisel plunger (1 mm diameter) delivered an occluso-gingival vertical force to the center of the interdental region of the retainer at room temperature. To simulate masticatory forces, the specimens were subjected to 10^6^ cyclic loadings,^16^ a pre-set load of 20 N,^17^ and at 2 Hz,^18^ corresponding to the estimated oral chewing frequency. Cyclic loading was applied either until the retainer failed or until the limit of cycles was reached. Any failure during the mechanical fatigue was recorded. 

### B. ACID CHALLENGE

Fifty bonded specimens were immersed in a 2.5 pH acidic solution of diluted hydrochloric acid (HCl) for a 5-minute session, three times daily, and separated by 2-hour intervals of re-immersion in distilled water (pH 6) at 37°C. The samples were then stored in distilled water for the rest of the day at 37°C. Before and after each session, samples were rinsed under running water and air-dried. After each session, the acidic solution and the distilled water were replaced with new, freshly prepared solutions. This protocol was repeated for thirty days^19^, to mimic the impact of intraoral acidity fluctuation. 

### C. DEBONDING PROCEDURE

**Figure 3:**
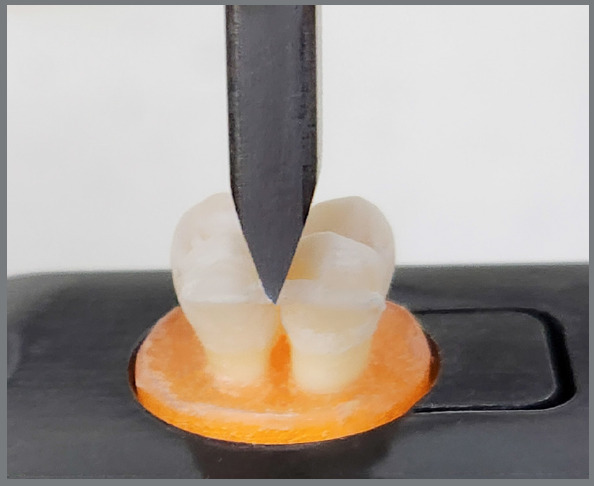
The debonding test, with the block fixed into the sample-holding tool and the plunger exerting a vertical load on the interdental region.

Following artificial aging and acid challenge, all blocks were debonded using a universal testing machine (Tinius Olsen; H50KT, England) equipped with a load cell of 50 kN at room temperature. The lower jaw of the machine was equipped with a sample-holding tool that was used to fix the sample individually. The upper moving jaw was supplied with a plunger with a diameter of 1 mm. The crosshead, imitating the force of bite, exerted a 1 mm/minute vertical load on the interdental midpoint. The applied load was raised gradually until failure happened on a minimum of one bonding site of the sample (Fig. 3). 

## FAILURE PATTERNS

Two operators investigated the enamel and wire surfaces in a stereomicroscope with 10x magnification, following the retainers’ failure. The examiner conducted assessments again after a month, and a different operator assessed the same specimens. The intra-examiner reliability and the inter-examiner reliability were verified using the Kappa test, which was evaluated according to the ratings of Landis and Koch.^20^ The results indicated a nearly perfect agreement (0.97 each). The failure patterns for the two bonding locations were recorded, and the data of the first to fail was analyzed. A smaller score was assigned in instances where the two bonding locations failed at the same time. The ranking system was altered from the one proposed by Foek et al.^16^, in the following manner:

» Type 1: indicates complete debonding of the adhesive from the tooth surface.
» Type 2: indicates partial debonding of the adhesive from the tooth surface.
» Type 3: indicates that the adhesive was not debonded from the tooth surface; rather, the covering adhesive over the retainer has detached.
» Type 4: indicates that the retainer was not debonded from the tooth surface; rather, it was fractured.
» Type 5: indicates that the adhesive was not debonded from the tooth surface; rather, the retainer has dislodged.

## STATISTICAL ANALYSES

The SPSS software program (version 25; IBM, USA) was used to conduct statistical analyses witha level of significance of p < 0.05. The reliability of failure mechanisms was evaluated using Kappa ratings. The debonding forces were subjected to the Shapiro-Wilk test and Levene’s test, which revealed that the data had a normal distribution, with identical variances. Thus, Tukey HSD *post-hoc* tests were implemented after the one-way ANOVA. The failure patterns were compared using the Kruskal-Wallis test, followed by the Pairwise comparison test.

## RESULTS

The specimens survived thermocycling, cyclic loading, and acid challenge. The 3D-printed semi-elliptical retainers yielded the greatest debonding forces for the two debonding tests, with the 3D-printed oval, G&H^®^ MSWs, 3D-printed round, and Respond^®^ MSWs retainers following, in that order. Debonding forces showed statistically significant differences in all comparisons across groups, according to Tukey HSD *post-hoc* multiple comparisons. However, no significant disparities existed between 3D-printed semi-elliptical and oval retainers following the acid challenge (Table 1).

**Table 1:** Debonding forces (N) of 3D-printed customized retainers versus conventional retainers.

Tests	Groups	n	Mean	SD	Min	Max	ANOVA	***Post-hoc*** **significance**
Debonding after aging	3D-printed round	10	84.310	2.250	81.50	88.30	F = 3017.411 df = 49 p = 0.000*	All groups*
	3D-printed oval	10	112.250	1.639	109.70	115.70		All groups*
	3D-printed semi-elliptical	10	114.310	1.051	112.80	115.90		All groups*
	G&H^®^ MSWs	10	107.330	1.179	105.50	109.00		All groups*
	Respond^®^ MSWs	10	50.740	1.310	48.50	52.50		All groups*
Debonding after acid challenge	3D-printed round	10	92.610	1.769	90.10	95.20	F = 1811.171 df = 49 p = 0.000*	All groups*
	3D-printed oval	10	120.910	2.409	116.50	124.50		3D-printed round* G&H^®^ MSWs* Respond^®^ MSWs*
	3D-printed semi-elliptical	10	122.680	2.426	118.50	125.60		3D-printed round* Respond^®^ MSWs* G&H^®^ MSWs*
	G&H^®^ MSWs	10	110.730	2.609	107.50	114.40		All groups*
	Respond^®^ MSWs	10	52.850	1.203	51.50	55.00		All groups*

* The difference is significant.

## FAILURE PATTERNS

Most of the 3D-printed retainers presented type 4 failure. G&H^®^ MSWs experienced types 2, 3, and 5, whereas type 5 failures predominated in Respond^®^ MSWs during both debonding tests. The Kruskal-Wallis test showed that retainer type affected failure patterns after artificial aging, but not after acid challenge. Pairwise comparisons after aging demonstrated significant differences for G&H^®^ MSWs compared to 3D-printed round retainers and Respond^®^ MSWs. The significance values were adjusted by Bonferroni correction (Table 2).

**Table 2:** Failure patterns of 3D-printed customized retainers versus conventional retainers.

Tests	Groups	n	Failure Patterns				Kruskal Wallis Statistics		Pairwise Significance
			Type 1	Type 2	Type 3	Type 4	Type 5		
Failure patterns after aging	3D-printed round	10	0	0	0	10	0	H = 16.114 df = 4 p-value = 0.003*	G&H^®^ MSWs*
	3D-printed oval	10	0	1	0	9	0		None
	3D-printed semi-elliptical	10	0	1	0	9	0		None
	G&H^®^ MSWs	10	0	7	2	0	1		3D-printed round * Respond^®^ MSWs *
	Respond^®^ MSWs	10	0	3	0	0	7		G&H^®^ MSWs*
Failure patterns after acid challenge	3D-printed round	10	0	1	0	9	0	H = 4.617 df = 4 p-value = 0.329	
	3D-printed oval	10	0	2	0	8	0		
	3D-printed semi-elliptical	10	0	2	0	8	0		
	G&H^®^ MSWs	10	0	2	3	0	5		
	Respond^®^ MSWs	10	0	3	0	0	7		

* The difference is significant.

## DISCUSSION

Bonded lingual retainers are supposed to function in harsh oral environments, including long-term humidity, fluctuations in pH and temperature, and cyclic loading caused by mastication, occlusion, and intraoral parafunctional habits. Lifelong retention burdens the lingual retainers’ durability.^2^ However, the main problem associated with MSWs is the high rate of retainer failure, particularly in the maxilla. This failure is primarily due to bond failure, which may be caused by masticatory forces and occlusal interferences.^1^ On the other hand, a real-life orthodontic patient frequently consumes a highly acidic beverage. This study verified the long-term durability of the bonding and the new 3D-printed retainers under the influence of artificial *in-vitro* aging and acid challenge models.

All specimens survived thermocycling, cyclic loading, and acid attack, suggesting that the retainers complex could tolerate these aggressive stresses and, consequently, predict clinical durability. The 3D-printed semi-elliptical and oval retainers achieved the greatest debonding forces. Their digital designs and flat shapes resulted in a more even distribution of forces over the teeth surfaces, and improved durability when exposed to bending stress across their bigger dimensions. Additionally, the chemical robust bonds with the adhesive as well as the nanofiller particles were assumed to give superior bond and strength, respectively. However, 3D-printed retainers were fractured rather than debonded, following both aging and acid attacks. Water exposure (during thermocycling and acid challenge) might attack the ester bonds in methacrylate, the major constituent of the 3D-printed resin and composite adhesive. Water particle diffusion into the matrix could initiate two series of events in the composite material, including hygroscopic expansion and chemical degradation.^21^ It could act as a plasticizer of the polymer, thereby deteriorating the structural integrity of resins, decreasing their mechanical performance, and accelerating the process of failure.^22^ The hydrolytic degradation process of the 3D-printed resin matrix may be attributed to the polarity of the oxane bonds connecting the filler and silanes; thus, the polymer microstructure is significantly altered and irreversibly damaged. Thermocycling can potentially increase water sorption, particularly in warm temperatures.^23^ The flexural strength of 3D-printed resins deteriorated associated with thermocycling^24^^,^^25^ or related to water storage.^26^^,^^27^ On the other hand, TEGDMA, marked by hydrophilic ether linkages, exhibits the highest hydrophilicity, followed by bis-GMA; both are the ingredients of the Transbond LR adhesive,^28^ yet the water sorption property of 3D-printed resin is thought to be more prominent, due to its microstructure. Additive manufacturing generates multiple layers; therefore, water permeates in micro-spaces and displaces the layers from one another.^22^ Considering the possible incomplete polymerization at the interface between layers, microporosities, and residual monomers can also contribute to the increased hydrolysis potential. Moreover, 3D-printed materials have relatively low nanofiller content, as opposed to Transbond LR (high filler, 75-85%), and the relationship between surface degradation and filler content is inverse.^29^ Conversely, 3D-printed retainers may enhance thermal stability and mitigate mechanical fatigue formation in the retainer-adhesive-enamel complex, in comparison to MSWs. The adverse effect of thermal fluctuation and cyclic loading on materials with comparable coefficients of thermal expansion, thermal conductivity, and physical properties is more predictable and has an equivalent impact.^16^ However, a uniaxial compression of the 3D-printed sample resulted in the formation of numerous microcracks, potentially extending from the main cracks created between the layers. Also, the defective resin matrix caused by water diffusion could gradually undermine the reinforcing effects of the nanofillers, weakening the corroded subsurface layer. The direct cyclic loading renders it more susceptible to contact wear,^30^ a significant contributor to the dental materials’ deterioration. Multiple preceding factors could contribute to the fracturing of 3D-printed retainers. On the contrary, the physical properties differences between metal MSWs and adhesive are more remarkable than between enamel and adhesive. Consequently, the wire-adhesive bond would weaken with mechanical fatigue. 

Notably, the 3D-printed oval and semi-elliptical retainers exhibited comparable debonding values for both debonding categories; although significant differences after aging signified different stress distribution patterns during cyclic loading. Additionally, the better adaptability of 3D-printed semi-elliptical retainers to the teeth’s lingual anatomy could potentially play a role. Nevertheless, these differences were not clinically noteworthy. This is indicative of their similar cross-sectional areas.

Bonding efficacy and the 3D-printed retainers’ strength could be compromised due to water sorption, erosive effects of the acidity, or both, resulting in degradation of the resin’s polymer network. Methacrylate degradation is known to be accelerated by pH variations.^31^ However, this process is impacted by additional intraorally variables, including the presence of enzymes and saliva’s buffering capacity, which mitigates the erosive effects of acids.

SprintRay OnX 3D-printed material was selected because, as stated by the manufacturer, it was designed to deliver superior flexural strength and modulus in its class, a critical consideration when utilizing a lingual retainer. Only one nanoceramic material was tested, which limits judgements about the overall efficiency of 3D-printed retainers. This research idea was not focused on a particular material; it was about the utilization of 3D printing for the fabrication of customized lingual retainers. In addition, the nanomaterials sector is always expanding and developing on a daily basis. Integrating nanotechnology and 3D printing can potentially customize the 3D printing material properties to meet certain clinical needs. This was shown by the material made with Nano-Fusion^™^ (OnX Tough), introduced by the same SprintRay company one year after the SprintRay OnX, with superior qualities. It guarantees denser polymer chains and more homogenous particle distribution. It might impact biomechanical behavior and, therefore, the longevity of 3D-printed retainers made on a manner similar to those employed in this study. 

For the lingual comfort of the patient, the retainers’ lingual aspects were designed with rounded designs. In addition to round wire, semi-elliptical and oval cross-sectional wires were set up, so that their highest dimension were across the lingual surfaces of the teeth, to increase their strength and the surface area of retention, yet its thickness was held to a minimum, which would have allowed for a lower profile, avoiding bulky constructions and improving patient comfort. Nonetheless, any cross-sectional shape may be designed and produced with 3D printing. In the present study, only three cross-sectional designs were investigated; and a broader range of cross-sectional designs might reveal additional information about the best shapes for 3D-printed retainers. 

The results of this study are inherently incapable of reproducing intricate clinical situations, as is the case with any *in-vitro* research. However, assessing debond forces following rigorous aging methods mitigated some of these variables. Clinical studies are required to evaluate the effect of the patient real mastication, saliva composition, physiologic teeth movement, tongue functional forces, mouth rinsing or brushing, and the presence of calculus and plaque, which may significantly impact the debonding process. 

## CONCLUSION

The 3D-printed nanoceramic hybrid semi-elliptical and oval retainers demonstrated desirable debonding forces, although the cohesive fracture pattern was dominant, following both artificial aging and acid challenge protocols. Nevertheless, long-term clinical studies are warranted.

## Data Availability

Due to the sensitive nature of the data and ethical restrictions, the datasets supporting this study are not publicly available.
